# Supplementary material for: The Molecular Epidemiology of Clade 2.3.4.4B H5N1 High Pathogenicity Avian Influenza in Southern Africa, 2021–2022
Source: Viruses. 2023 Jun 16;15(6):1383. doi: 10.3390/v15061383 (PMC10302261; doi:10.3390/v15061383)
Supplement: Supplementary file 1 [file viruses-15-01383-s001.zip › Figure S1a¿Ch.pdf]

Figure S1.(a) to (h).

1(a)

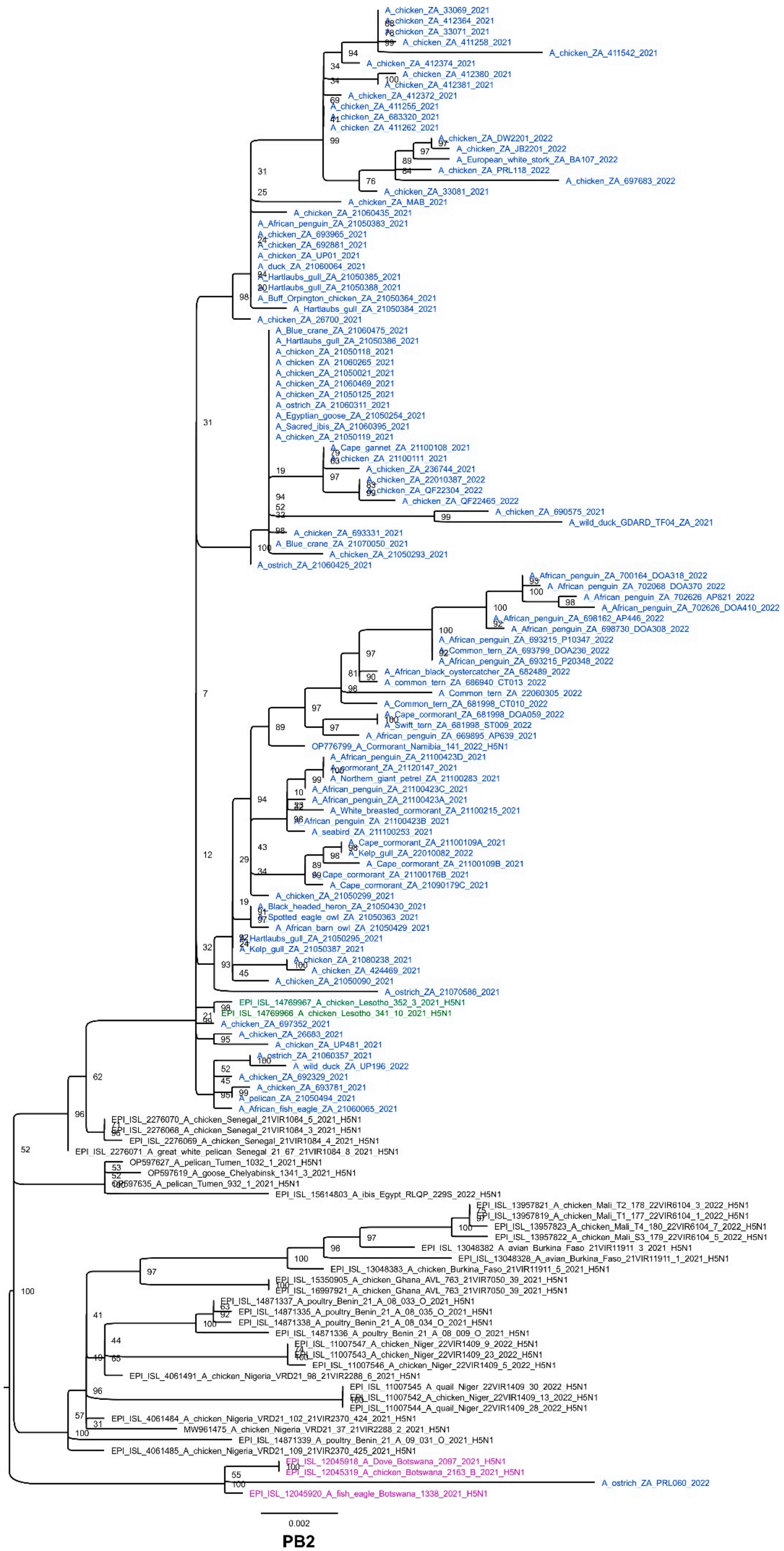

PB2

1(b)

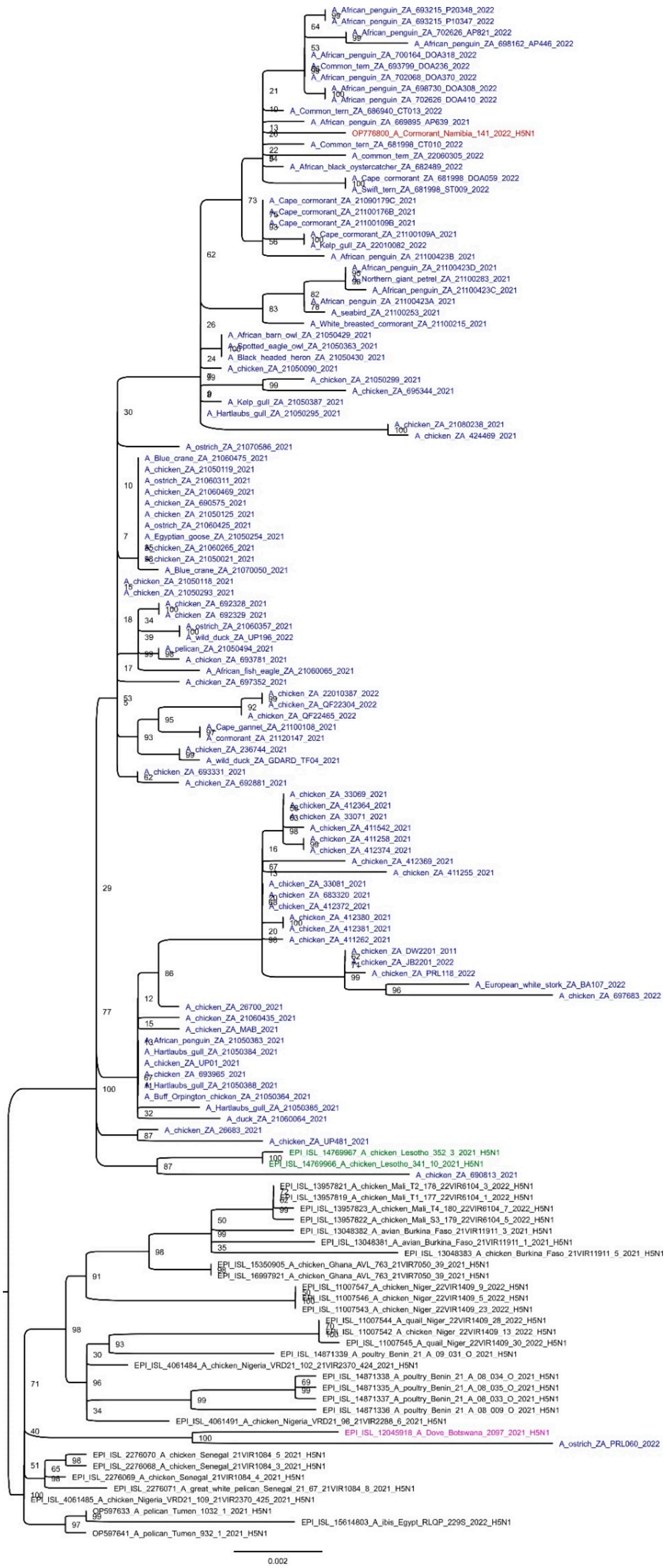

PB1

1(c)

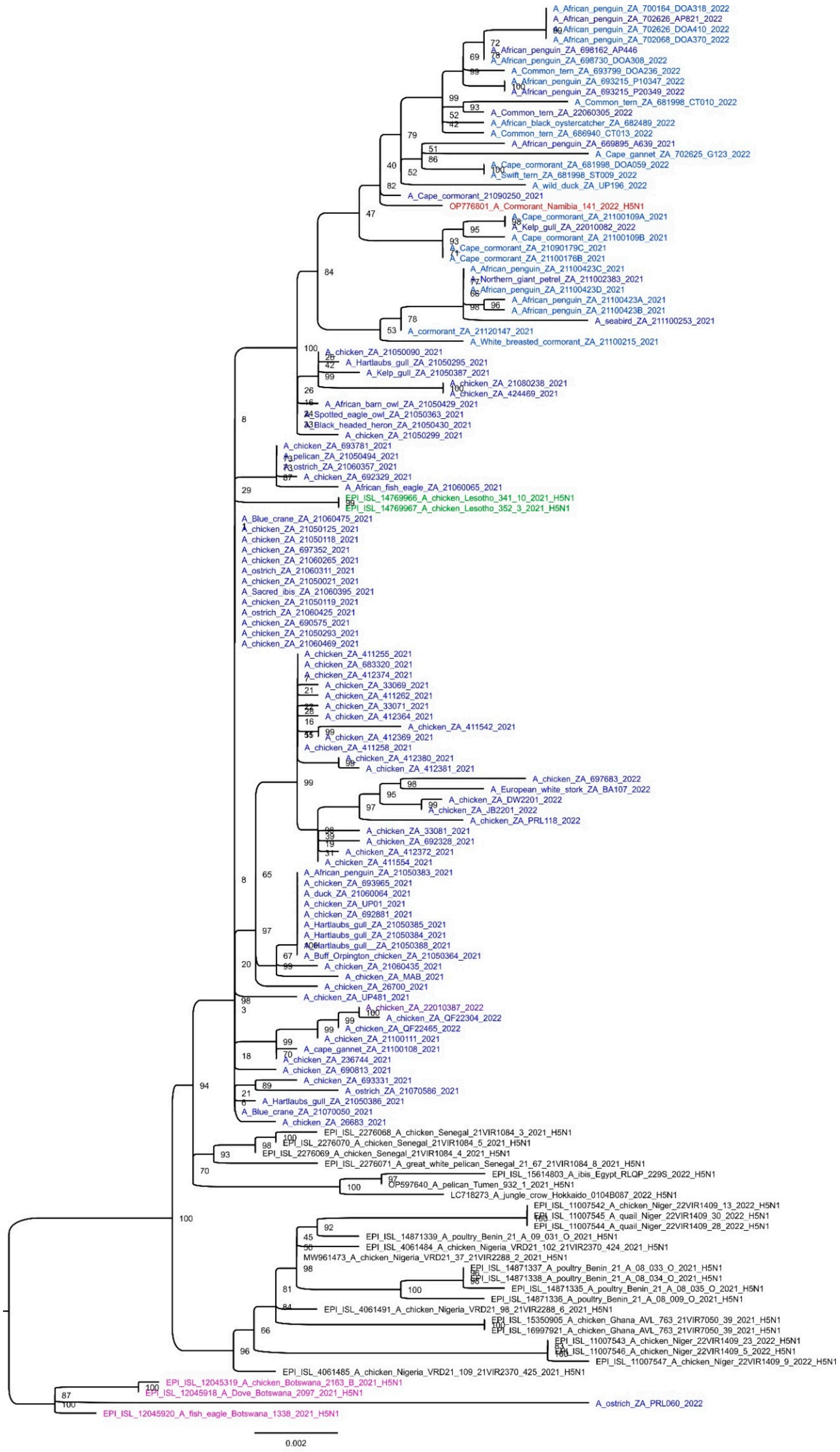

PA

1(d)

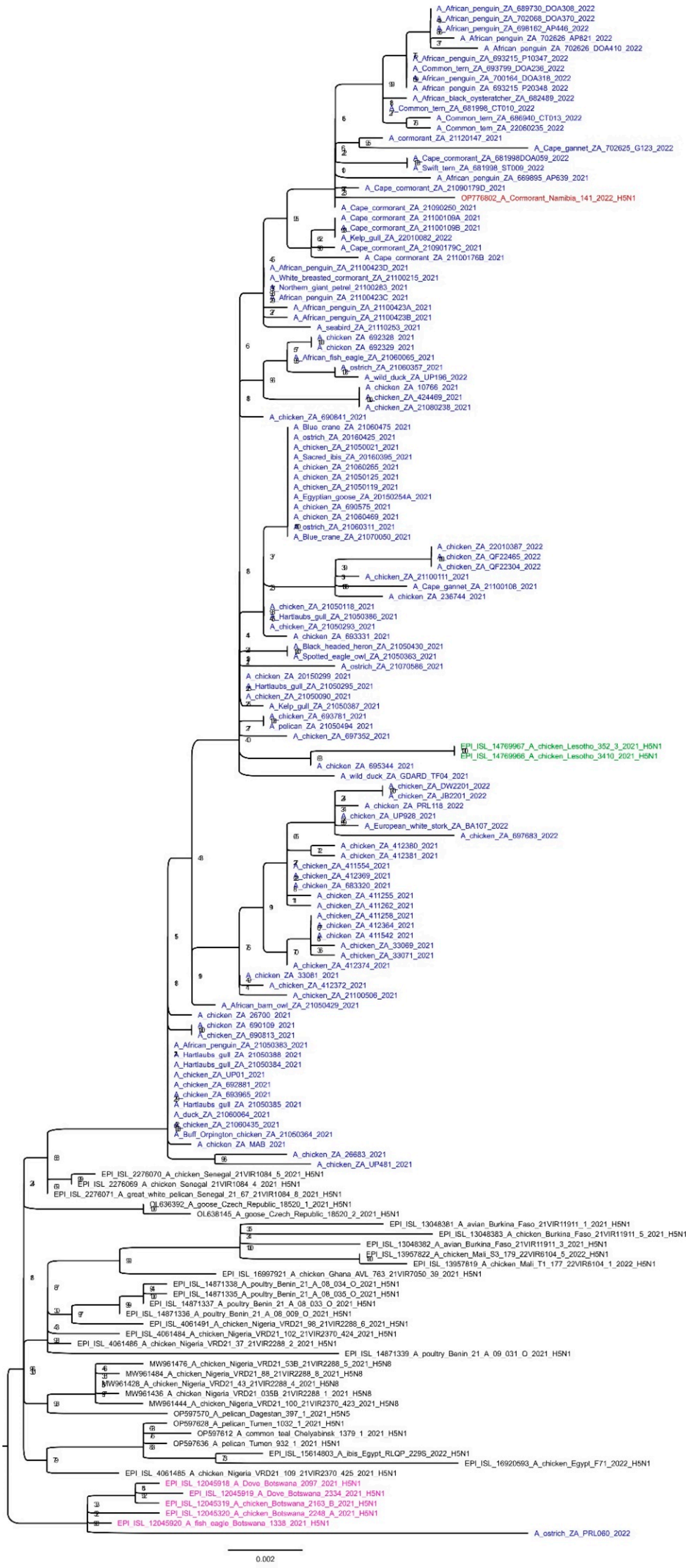

HA

1(e)

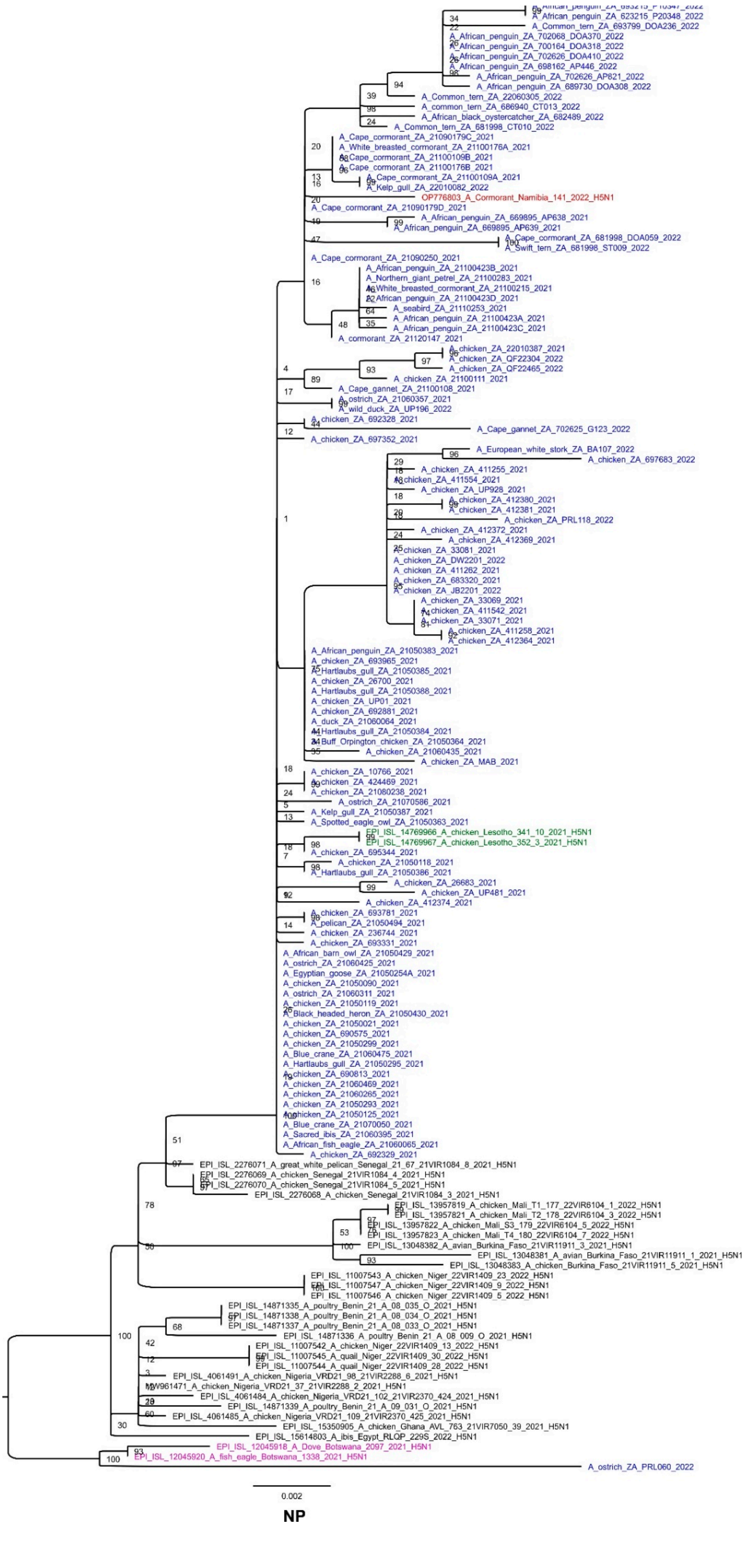

NP

1(f)

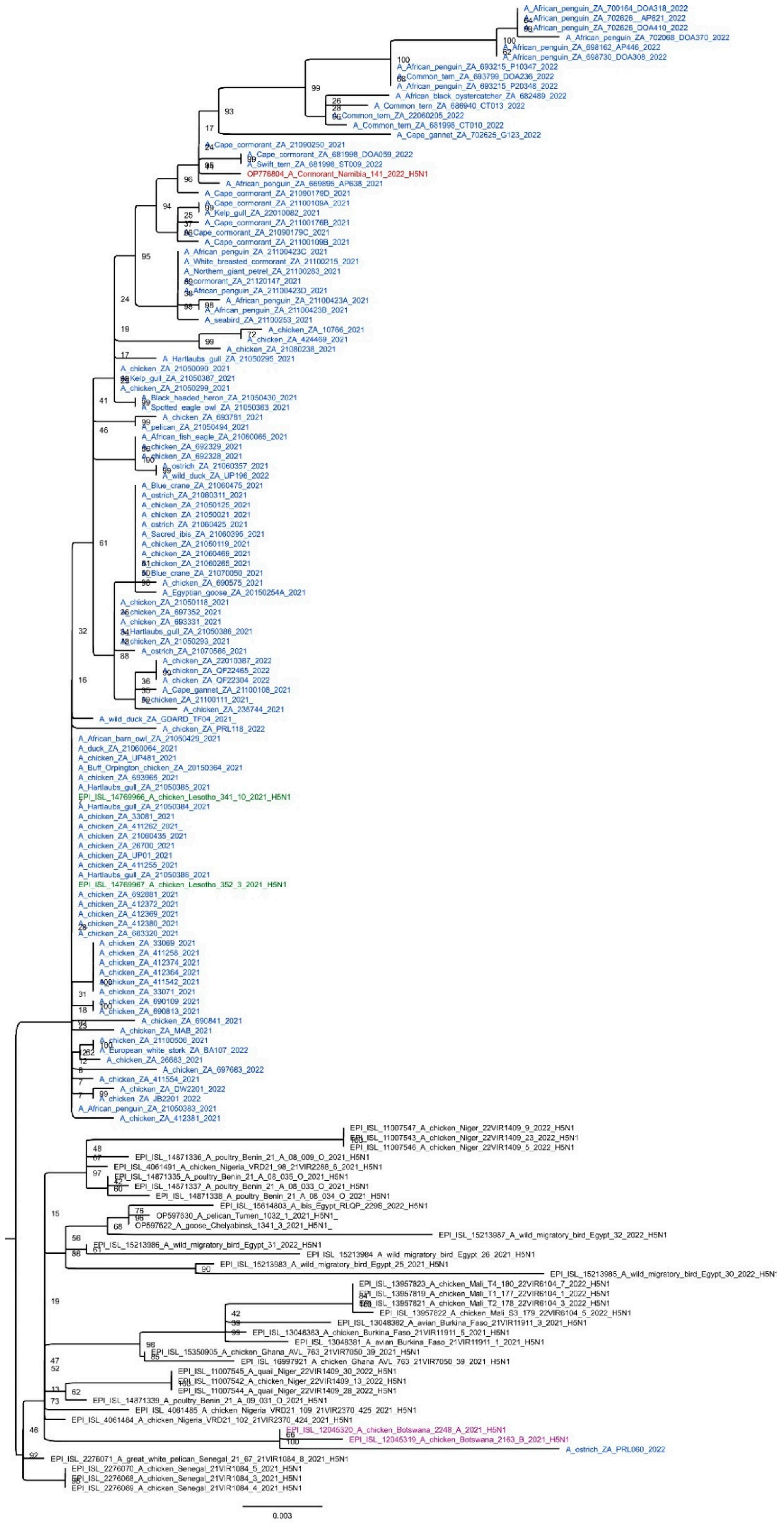

A_African_penguin_ZA_700164_DOA318_2022
A_African_penguin_ZA_702626__AP821_2022
A_African_penguin_ZA_702626_DOA410_2022
A_African_penguin_ZA_702068_DOA370_2022
A_African_penguin_ZA_698162_AP446_2022
A_African_penguin_ZA_698730_DOA308_2022
A_African_penguin_ZA_693215_P10347_2022
A_Common_tern_ZA_693799_DOA236_2022
A_African_penguin_ZA_693215_P20348_2022
A_African_black_oystercatcher_ZA_682489_2022
A_Common_tern_ZA_686940_CT013_2022
A_Common_tern_ZA_22060205_2022
A_Common_tern_ZA_681998_CT010_2022
A_Cape_gannet_ZA_702625_G123_2022
A_Cape_cormorant_ZA_21090250_2021
A_Cape_cormorant_ZA_681998_DOA059_2022
A_Swift_tern_ZA_681998_ST009_2022
OP776804_A_Cormorant_Namibia_141_2022_H5N1
A_African_penguin_ZA_669895_AP638_2021
A_Cape_cormorant_ZA_21090179D_2021
A_Cape_cormorant_ZA_21100109A_2021
A_Kelp_gull_ZA_22010082_2021
A_Cape_cormorant_ZA_21100176B_2021
A_Cape_cormorant_ZA_21090179C_2021
A_Cape_cormorant_ZA_21100109B_2021
A_African_penguin_ZA_21100423C_2021
A_White_breasted_cormorant_ZA_21100215_2021
A_Northern_giant_petrel_ZA_21100283_2021
A_cormorant_ZA_21120147_2021
A_African_penguin_ZA_21100423D_2021
A_African_penguin_ZA_21100423A_2021
A_African_penguin_ZA_21100423B_2021
A_seabird_ZA_21100253_2021
A_chicken_ZA_10766_2021
A_chicken_ZA_424469_2021
A_chicken_ZA_21080238_2021
A_Hartlaubs_gull_ZA_21050295_2021
A_chicken_ZA_21050090_2021
A_Kelp_gull_ZA_21050387_2021
A_chicken_ZA_21050299_2021
A_Black_headed_heron_ZA_21050430_2021
A_Spotted_eagle_owl_ZA_21050363_2021
A_chicken_ZA_693781_2021
A_pelican_ZA_21050494_2021
A_African_fish_eagle_ZA_21060065_2021
A_chicken_ZA_692329_2021
A_chicken_ZA_692328_2021
A_ostrich_ZA_21060357_2021
A_wild_duck_ZA_UP196_2022
A_Blue_crane_ZA_21060475_2021
A_ostrich_ZA_21060311_2021
A_chicken_ZA_21050125_2021
A_chicken_ZA_21050021_2021
A_ostrich_ZA_21060425_2021
A_Sacred_ibis_ZA_21060395_2021
A_chicken_ZA_21050119_2021
A_chicken_ZA_21060469_2021
A_chicken_ZA_21060265_2021
A_Blue_crane_ZA_21070050_2021
A_chicken_ZA_690575_2021
A_Egyptian_goose_ZA_20150254A_2021
A_chicken_ZA_21050118_2021
A_chicken_ZA_697352_2021
A_chicken_ZA_693331_2021
A_Hartlaubs_gull_ZA_21050386_2021
A_chicken_ZA_21050293_2021
A_ostrich_ZA_21070586_2021
A_chicken_ZA_22010387_2022
A_chicken_ZA_QF22465_2022
A_chicken_ZA_QF22304_2022
A_Cape_gannet_ZA_21100108_2021
A_chicken_ZA_21100111_2021_
A_chicken_ZA_236744_2021
A_wild_duck_ZA_GDARD_TF04_2021_
A_chicken_ZA_PRL118_2022
A_African_barn_owl_ZA_21050429_2021
A_duck_ZA_21060064_2021
A_chicken_ZA_UP481_2021
A_Buff_Orpington_chicken_ZA_20150364_2021
A_chicken_ZA_693965_2021
A_Hartlaubs_gull_ZA_21050385_2021
EPI_ISL_14769966_A_chicken_Lesotho_341_10_2021_H5N1
A_Hartlaubs_gull_ZA_21050384_2021
A_chicken_ZA_33081_2021
A_chicken_ZA_411262_2021_
A_chicken_ZA_21060435_2021
A_chicken_ZA_26700_2021
A_chicken_ZA_UP01_2021
A_chicken_ZA_411255_2021
A_Hartlaubs_gull_ZA_21050388_2021
EPI_ISL_14769967_A_chicken_Lesotho_352_3_2021_H5N1
A_chicken_ZA_692881_2021
A_chicken_ZA_412372_2021
A_chicken_ZA_412369_2021
A_chicken_ZA_412380_2021
A_chicken_ZA_683320_2021
A_chicken_ZA_33069_2021
A_chicken_ZA_411258_2021
A_chicken_ZA_412374_2021
A_chicken_ZA_412364_2021
A_chicken_ZA_411542_2021
A_chicken_ZA_33071_2021
A_chicken_ZA_690109_2021
A_chicken_ZA_690813_2021
A_chicken_ZA_690841_2021
A_chicken_ZA_MAB_2021
A_chicken_ZA_21100506_2021
A_European_white_stork_ZA_BA107_2022
A_chicken_ZA_26683_2021
A_chicken_ZA_697683_2022
A_chicken_ZA_411554_2021
A_chicken_ZA_DW2201_2022
A_chicken_ZA_JB2201_2022
A_African_penguin_ZA_21050383_2021
A_chicken_ZA_412381_2021
EPI_ISL_11007547_A_chicken_Niger_22VIR1409_9_2022_H5N1
EPI_ISL_11007543_A_chicken_Niger_22VIR1409_23_2022_H5N1
EPI_ISL_11007546_A_chicken_Niger_22VIR1409_5_2022_H5N1
EPI_ISL_14871336_A_poultry_Benin_21_A_08_009_O_2021_H5N1
EPI_ISL_4061491_A_chicken_Nigeria_VRD21_98_21VIR2288_6_2021_H5N1
EPI_ISL_14871335_A_poultry_Benin_21_A_08_035_O_2021_H5N1
EPI_ISL_14871337_A_poultry_Benin_21_A_08_033_O_2021_H5N1
EPI_ISL_14871338_A_poultry_Benin_21_A_08_034_O_2021_H5N1
EPI_ISL_15614803_A_ibis_Egypt_RLQP_229S_2022_H5N1
OP597630_A_pelican_Tumen_1032_1_2021_H5N1_
OP597622_A_goose_Chelyabinsk_1341_3_2021_H5N1_
EPI_ISL_15213987_A_wild_migratory_bird_Egypt_32_2022_H5N1
EPI_ISL_15213986_A_wild_migratory_bird_Egypt_31_2022_H5N1
EPI_ISL_15213984_A_wild_migratory_bird_Egypt_26_2021_H5N1
EPI_ISL_15213983_A_wild_migratory_bird_Egypt_25_2021_H5N1
EPI_ISL_15213985_A_wild_migratory_bird_Egypt_30_2022_H5N1
EPI_ISL_13957823_A_chicken_Mali_T4_180_22VIR6104_7_2022_H5N1
EPI_ISL_13957819_A_chicken_Mali_T1_177_22VIR6104_1_2022_H5N1
EPI_ISL_13957821_A_chicken_Mali_T2_178_22VIR6104_3_2022_H5N1
EPI_ISL_13957822_A_chicken_Mali_S3_179_22VIR6104_5_2022_H5N1
EPI_ISL_13048382_A_avian_Burkina_Faso_21VIR11911_3_2021_H5N1
EPI_ISL_13048383_A_chicken_Burkina_Faso_21VIR11911_5_2021_H5N1
EPI_ISL_13048381_A_avian_Burkina_Faso_21VIR11911_1_2021_H5N1
EPI_ISL_15350905_A_chicken_Ghana_AVL_763_21VIR7050_39_2021_H5N1
EPI_ISL_16997921_A_chicken_Ghana_AVL_763_21VIR7050_39_2021_H5N1
EPI_ISL_11007545_A_quail_Niger_22VIR1409_30_2022_H5N1
EPI_ISL_11007542_A_chicken_Niger_22VIR1409_13_2022_H5N1
EPI_ISL_11007544_A_quail_Niger_22VIR1409_28_2022_H5N1
EPI_ISL_14871339_A_poultry_Benin_21_A_09_031_O_2021_H5N1
EPI_ISL_4061485_A_chicken_Nigeria_VRD21_109_21VIR2370_425_2021_H5N1
EPI_ISL_4061484_A_chicken_Nigeria_VRD21_102_21VIR2370_424_2021_H5N1
EPI_ISL_12045320_A_chicken_Botswana_2248_A_2021_H5N1
EPI_ISL_12045319_A_chicken_Botswana_2163_B_2021_H5N1
A_ostrich_ZA_PRL060_2022
EPI_ISL_2276071_A_great_white_pelican_Senegal_21_67_21VIR1084_8_2021_H5N1
EPI_ISL_2276070_A_chicken_Senegal_21VIR1084_5_2021_H5N1
EPI_ISL_2276068_A_chicken_Senegal_21VIR1084_3_2021_H5N1
EPI_ISL_2276069_A_chicken_Senegal_21VIR1084_4_2021_H5N1
0.003

NA

1(g)

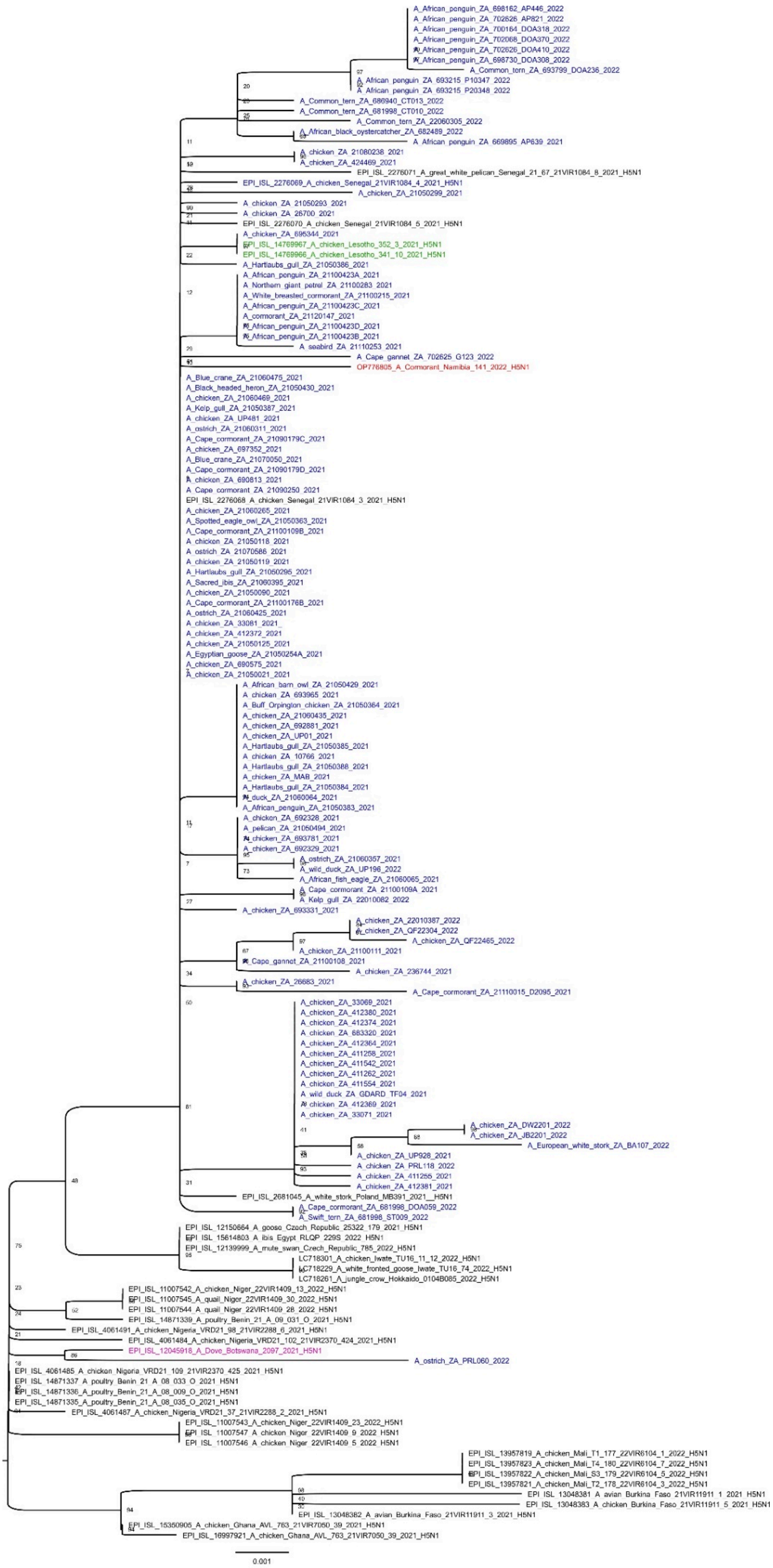
A_African_penguin_ZA_698162_AP446_2022
A_African_penguin_ZA_702626_AP821_2022
A_African_penguin_ZA_700164_DOA318_2022
A_African_penguin_ZA_702068_DOA370_2022
A_African_penguin_ZA_702626_DOA410_2022
A_African_penguin_ZA_698730_DOA308_2022
A_Common_tern_ZA_693799_DOA236_2022
A_African_penguin_ZA_693215_P10347_2022
A_African_penguin_ZA_693215_P20348_2022
A_Common_tern_ZA_686940_CT013_2022
A_Common_tern_ZA_681998_CT010_2022
A_Common_tern_ZA_22060305_2022
A_African_black_oystercatcher_ZA_682489_2022
A_African_penguin_ZA_669895_AP639_2021
A_chicken_ZA_21080238_2021
A_chicken_ZA_424469_2021
EPI_ISL_2276071_A_great_white_pelican_Senegal_21_67_21VIR1084_8_2021_H5N1
EPI_ISL_2276069_A_chicken_Senegal_21VIR1084_4_2021_H5N1
A_chicken_ZA_21050299_2021
A_chicken_ZA_21050293_2021
A_chicken_ZA_26700_2021
EPI_ISL_2276070_A_chicken_Senegal_21VIR1084_5_2021_H5N1
A_chicken_ZA_695344_2021
EPI_ISL_14769967_A_chicken_Lesotho_352_3_2021_H5N1
EPI_ISL_14769966_A_chicken_Lesotho_341_10_2021_H5N1
A_Hartlaubs_gull_ZA_21050386_2021
A_African_penguin_ZA_21100423A_2021
A_Northern_giant_petrel_ZA_21100283_2021
A_White_breasted_cormorant_ZA_21100215_2021
A_African_penguin_ZA_21100423C_2021
A_cormorant_ZA_21120147_2021
A_African_penguin_ZA_21100423D_2021
A_African_penguin_ZA_21100423B_2021
A_seabird_ZA_21110253_2021
A_Cape_gannet_ZA_702625_G123_2022
OP776805_A_Cormorant_Namibia_141_2022_H5N1
A_Blue_crane_ZA_21060475_2021
A_Black_headed_heron_ZA_21050430_2021
A_chicken_ZA_21060469_2021
A_Kelp_gull_ZA_21050387_2021
A_chicken_ZA_UP481_2021
A_ostrich_ZA_21060311_2021
A_Cape_cormorant_ZA_21090179C_2021
A_chicken_ZA_697352_2021
A_Blue_crane_ZA_21070050_2021
A_Cape_cormorant_ZA_21090179D_2021
A_chicken_ZA_690813_2021
A_Cape_cormorant_ZA_21090250_2021
EPI_ISL_2276068_A_chicken_Senegal_21VIR1084_3_2021_H5N1
A_chicken_ZA_21060265_2021
A_Spotted_eagle_owl_ZA_21050363_2021
A_Cape_cormorant_ZA_21100109B_2021
A_chicken_ZA_21050118_2021
A_ostrich_ZA_21070586_2021
A_chicken_ZA_21050119_2021
A_Hartlaubs_gull_ZA_21050295_2021
A_Sacred_ibis_ZA_21060395_2021
A_chicken_ZA_21050090_2021
A_Cape_cormorant_ZA_21100176B_2021
A_ostrich_ZA_21060425_2021
A_chicken_ZA_33081_2021
A_chicken_ZA_412372_2021
A_chicken_ZA_21050125_2021
A_Egyptian_goose_ZA_21050254A_2021
A_chicken_ZA_690575_2021
A_chicken_ZA_21050021_2021
A_African_barn_owl_ZA_21050429_2021
A_chicken_ZA_693965_2021
A_Buff_Orpington_chicken_ZA_21050364_2021
A_chicken_ZA_21060435_2021
A_chicken_ZA_692881_2021
A_chicken_ZA_UP01_2021
A_Hartlaubs_gull_ZA_21050385_2021
A_chicken_ZA_10766_2021
A_Hartlaubs_gull_ZA_21050388_2021
A_chicken_ZA_MAB_2021
A_Hartlaubs_gull_ZA_21050384_2021
A_duck_ZA_21060064_2021
A_African_penguin_ZA_21050383_2021
A_chicken_ZA_692328_2021
A_pelican_ZA_21050494_2021
A_chicken_ZA_693781_2021
A_chicken_ZA_692329_2021
A_ostrich_ZA_21060357_2021
A_wild_duck_ZA_UP196_2022
A_African_fish_eagle_ZA_21060065_2021
A_Cape_cormorant_ZA_21100109A_2021
A_Kelp_gull_ZA_22010082_2022
A_chicken_ZA_693331_2021
A_chicken_ZA_22010387_2022
A_chicken_ZA_QF22304_2022
A_chicken_ZA_QF22465_2022
A_chicken_ZA_21100111_2021
A_Cape_gannet_ZA_21100108_2021
A_chicken_ZA_236744_2021
A_chicken_ZA_26683_2021
A_Cape_cormorant_ZA_21110015_D2095_2021
A_chicken_ZA_33069_2021
A_chicken_ZA_412380_2021
A_chicken_ZA_412374_2021
A_chicken_ZA_683320_2021
A_chicken_ZA_412364_2021
A_chicken_ZA_411258_2021
A_chicken_ZA_411542_2021
A_chicken_ZA_411262_2021
A_chicken_ZA_411554_2021
A_wild_duck_ZA_GDARD_TF04_2021
A_chicken_ZA_412369_2021
A_chicken_ZA_33071_2021
A_chicken_ZA_DW2201_2022
A_chicken_ZA_JB2201_2022
A_European_white_stork_ZA_BA107_2022
A_chicken_ZA_UP928_2021
A_chicken_ZA_PRL118_2022
A_chicken_ZA_411255_2021
A_chicken_ZA_412381_2021
EPI_ISL_2681045_A_white_stork_Poland_MB391_2021__H5N1
A_Cape_cormorant_ZA_681998_DOA059_2022
A_Swift_tern_ZA_681998_ST009_2022
EPI_ISL_12150664_A_goose_Czech_Republic_25322_179_2021_H5N1
EPI_ISL_15614803_A_ibis_Egypt_RLQP_229S_2022_H5N1
EPI_ISL_12139999_A_mute_swan_Czech_Republic_785_2022_H5N1
LC718301_A_chicken_Iwate_TU16_11_12_2022_H5N1
LC718229_A_white_fronted_goose_Iwate_TU16_74_2022_H5N1
LC718261_A_jungle_crow_Hokkaido_0104B085_2022_H5N1
EPI_ISL_11007542_A_chicken_Niger_22VIR1409_13_2022_H5N1
EPI_ISL_11007545_A_quail_Niger_22VIR1409_30_2022_H5N1
EPI_ISL_11007544_A_quail_Niger_22VIR1409_28_2022_H5N1
EPI_ISL_14871339_A_poultry_Benin_21_A_09_031_O_2021_H5N1
EPI_ISL_4061491_A_chicken_Nigeria_VRD21_98_21VIR2288_6_2021_H5N1
EPI_ISL_4061484_A_chicken_Nigeria_VRD21_102_21VIR2370_424_2021_H5N1
EPI_ISL_12045918_A_Dove_Botswana_2097_2021_H5N1
A_ostrich_ZA_PRL060_2022
EPI_ISL_4061485_A_chicken_Nigeria_VRD21_109_21VIR2370_425_2021_H5N1
EPI_ISL_14871337_A_poultry_Benin_21_A_08_033_O_2021_H5N1
EPI_ISL_14871336_A_poultry_Benin_21_A_08_009_O_2021_H5N1
EPI_ISL_14871335_A_poultry_Benin_21_A_08_035_O_2021_H5N1
EPI_ISL_4061487_A_chicken_Nigeria_VRD21_37_21VIR2288_2_2021_H5N1
EPI_ISL_11007543_A_chicken_Niger_22VIR1409_23_2022_H5N1
EPI_ISL_11007547_A_chicken_Niger_22VIR1409_9_2022_H5N1
EPI_ISL_11007546_A_chicken_Niger_22VIR1409_5_2022_H5N1
EPI_ISL_13957819_A_chicken_Mali_T1_177_22VIR6104_1_2022_H5N1
EPI_ISL_13957823_A_chicken_Mali_T4_180_22VIR6104_7_2022_H5N1
EPI_ISL_13957822_A_chicken_Mali_S3_179_22VIR6104_5_2022_H5N1
EPI_ISL_13957821_A_chicken_Mali_T2_178_22VIR6104_3_2022_H5N1
EPI_ISL_13048381_A_avian_Burkina_Faso_21VIR11911_1_2021_H5N1
EPI_ISL_13048383_A_chicken_Burkina_Faso_21VIR11911_5_2021_H5N1
EPI_ISL_13048382_A_avian_Burkina_Faso_21VIR11911_3_2021_H5N1
EPI_ISL_15350905_A_chicken_Ghana_AVL_763_21VIR7050_39_2021_H5N1
EPI_ISL_16997921_A_chicken_Ghana_AVL_763_21VIR7050_39_2021_H5N1
0.001

M

1(h)

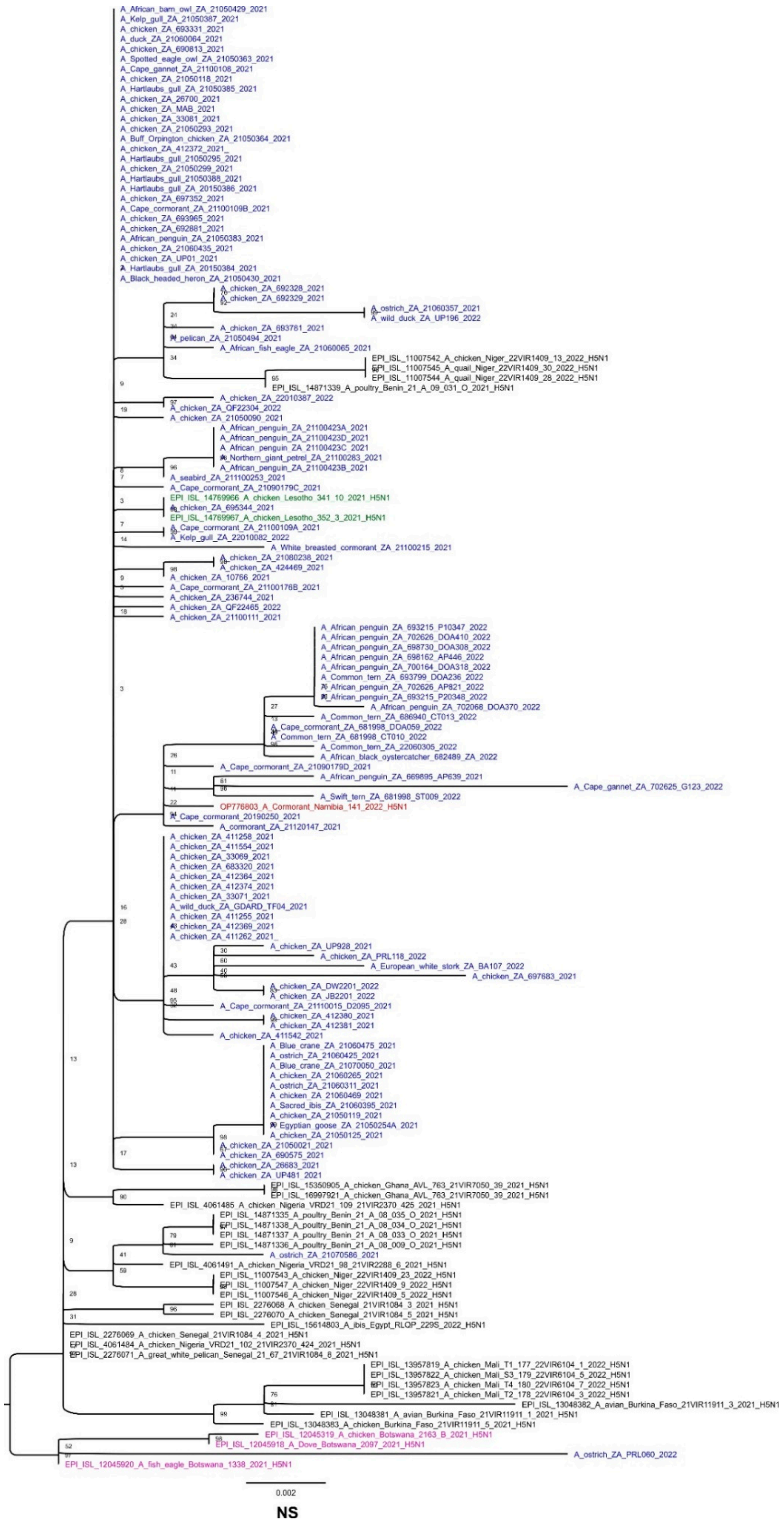

Supplemental Figure S1a–h. Maximum Likelihood phylogenetic trees of the individual genome segments of H5N1 HPAI viruses. All trees are midpoint-rooted and bootstrap values are indicated. South African viruses are highlighted in blue, Namibia in red, Lesotho in green, and Botswana in magenta.
