# Supplementary material for: The Molecular Epidemiology of Clade 2.3.4.4B H5N1 High Pathogenicity Avian Influenza in Southern Africa, 2021–2022
Source: Viruses. 2023 Jun 16;15(6):1383. doi: 10.3390/v15061383 (PMC10302261; doi:10.3390/v15061383)
Supplement: Supplementary file 1 [file viruses-15-01383-s001.zip › Figure S3a¿Ch.pdf]

Figure S3.(a) to (h).

3(a).

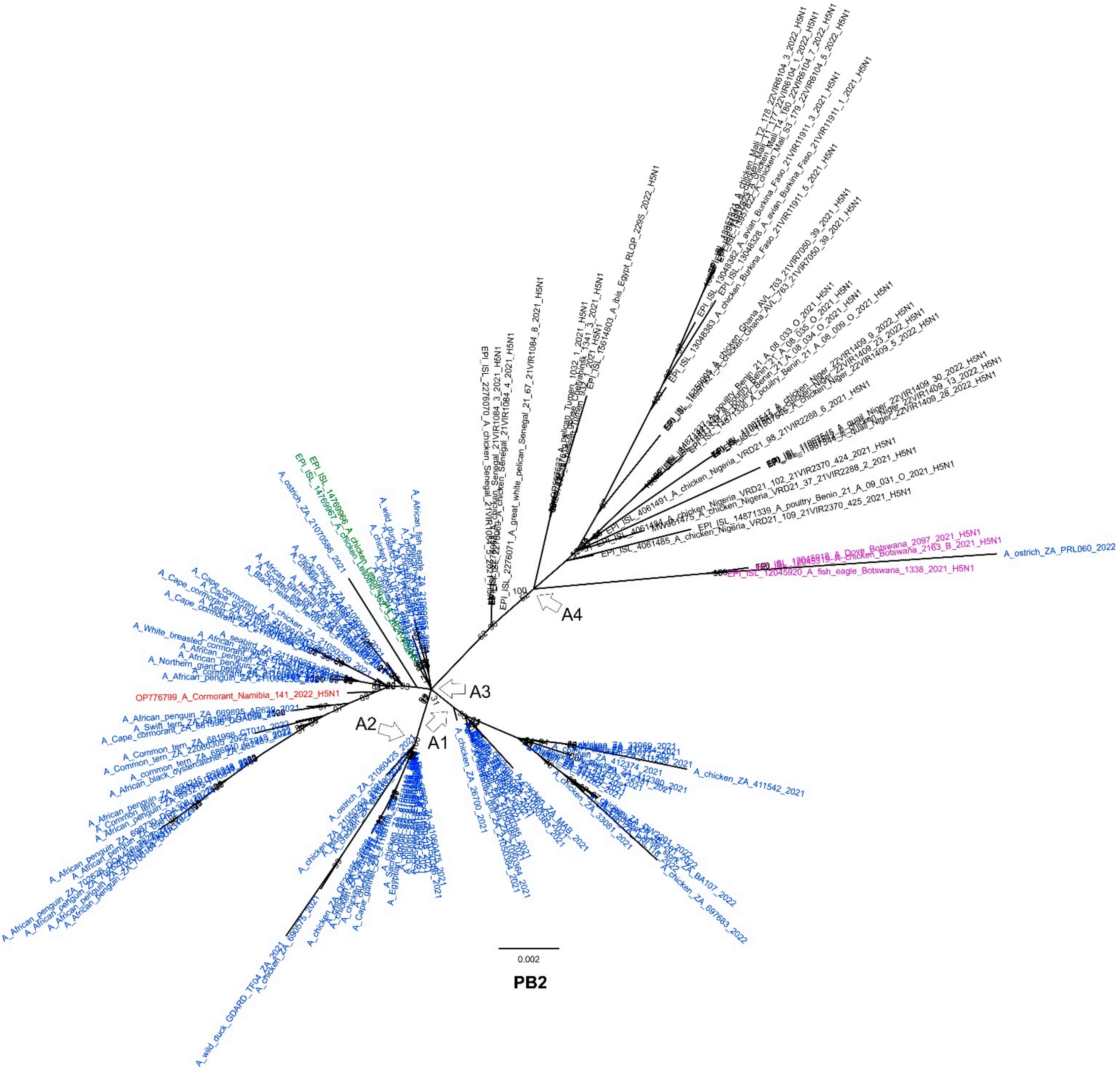

3(b).

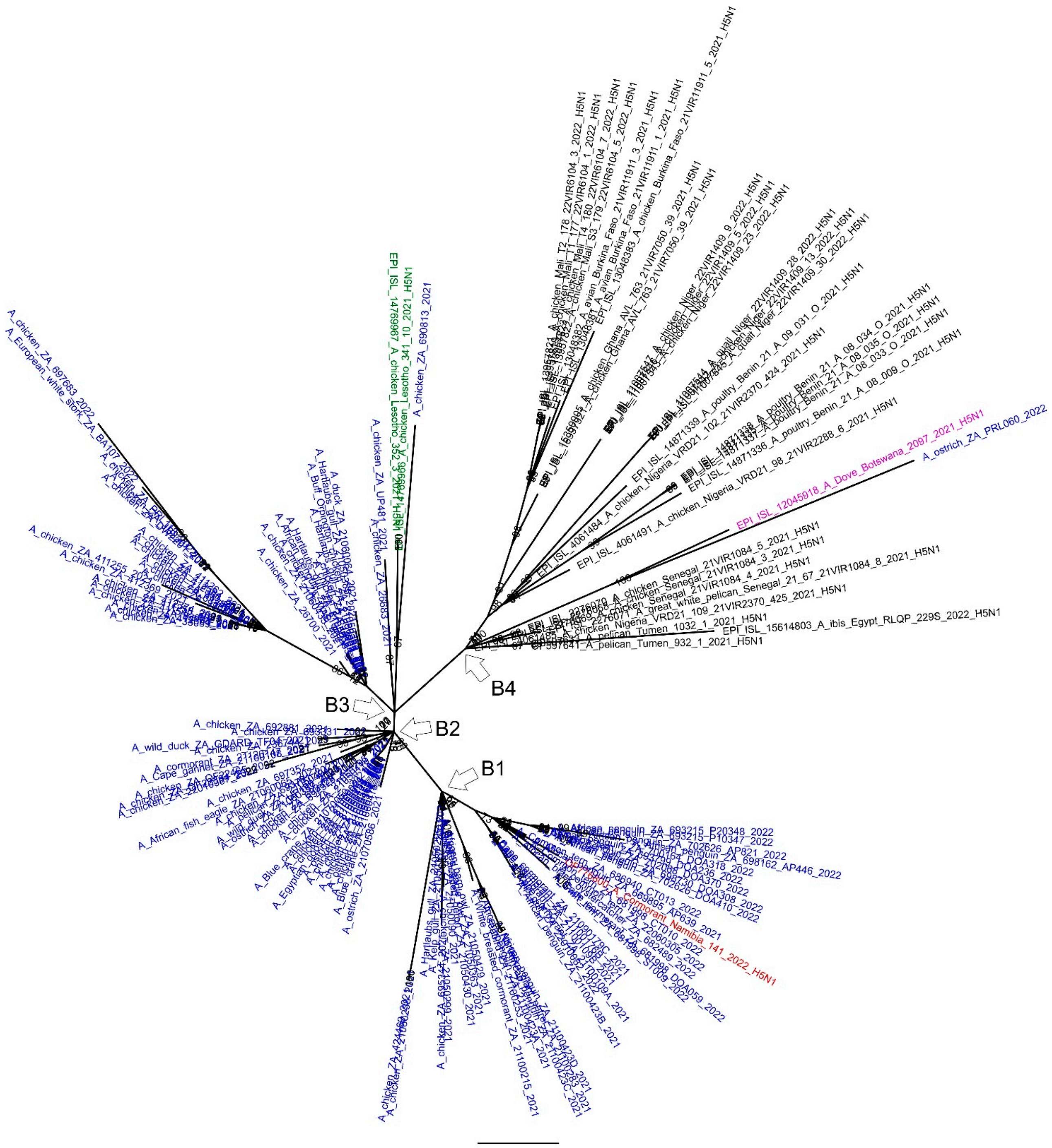
B1
B2
B3
B4
A_ostrich_ZA_PRL060_2022
EPI_ISL_12045918_A_Dove_Botswana_2097_2021_H5N1
EPI_ISL_15614803_A_ibis_Egypt_RLQP_229S_2022_H5N1
A_chicken_ZA_690813_2021
A_chicken_ZA_692881_2021
0.002

PB1

3(c).

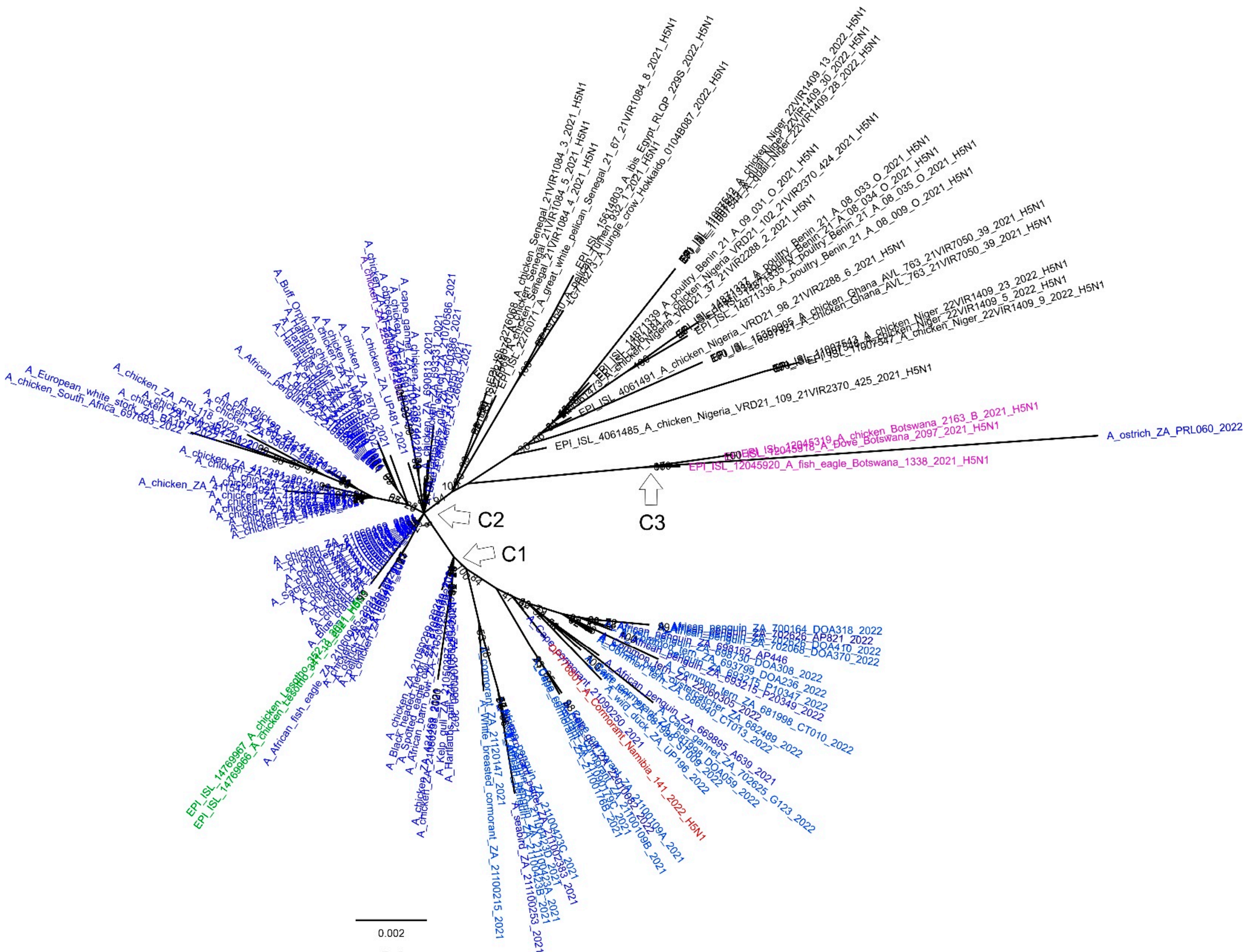
C1
C2
C3
0.002
A_ostrich_ZA_PRL060_2022
EPI_ISL_4061485_A_chicken_Nigeria_VRD21_109_21VIR2370_425_2021_H5N1
EPI_ISL_12045920_A_fish_eagle_Botswana_1338_2021_H5N1
A_chicken_South_Africa_697683_2022
A_chicken_Nigeria_VRD21_98_21VIR2288_6_2021_H5N1
cormorant_ZA_21100215_2021

PA

3(d).

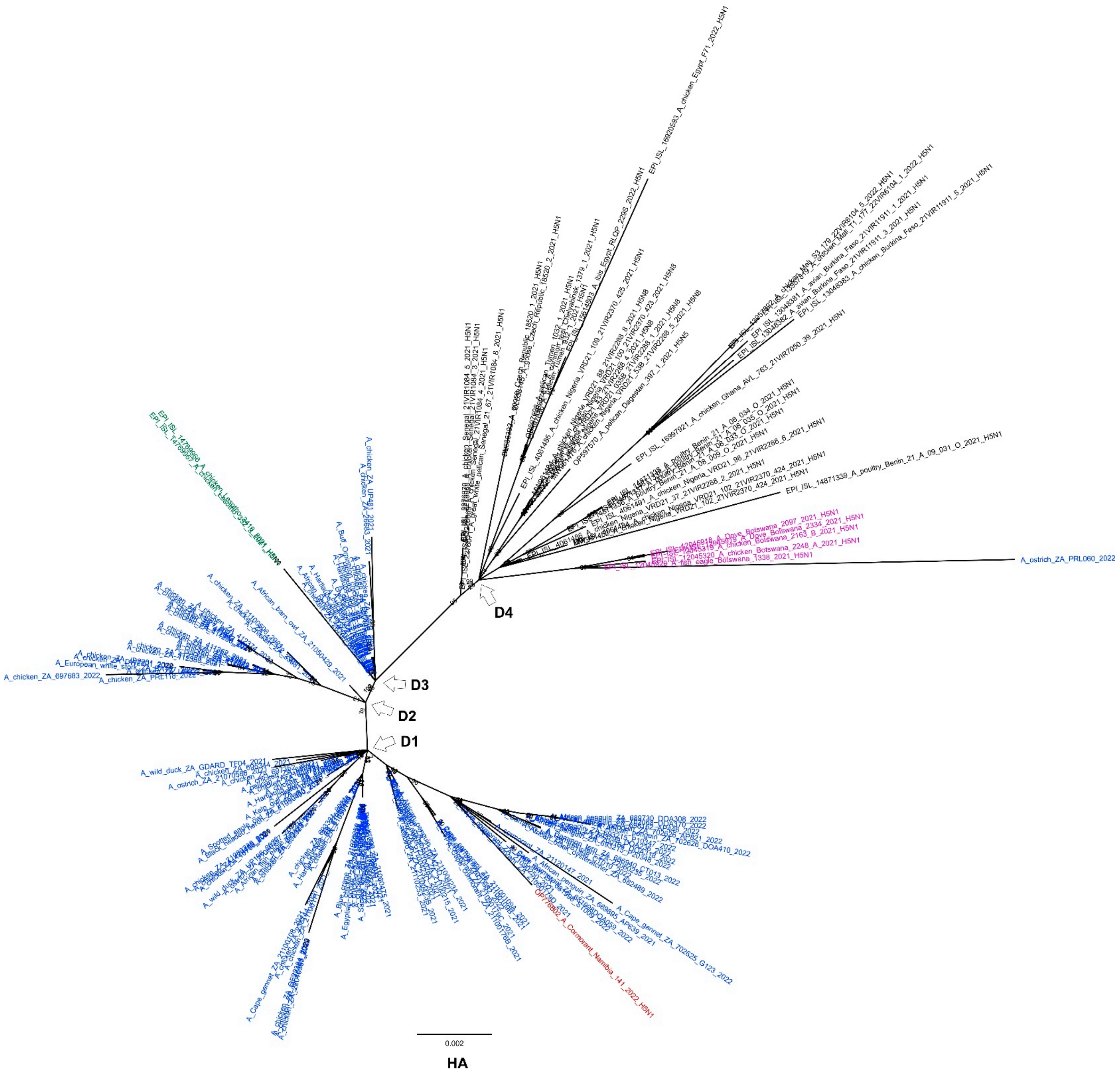

D1
D2
D3
D4
A_ostrich_ZA_PRL060_2022
A_chicken_ZA_697683_2022
EPI_ISL_16920593_A_chicken_Egypt_F71_2022_H5N1
OP776892_A_Cormorant_Namibia_141_2022_H5N1
A_Cape_gannet_ZA_702625_G123_2022
0.002
HA

3(e).

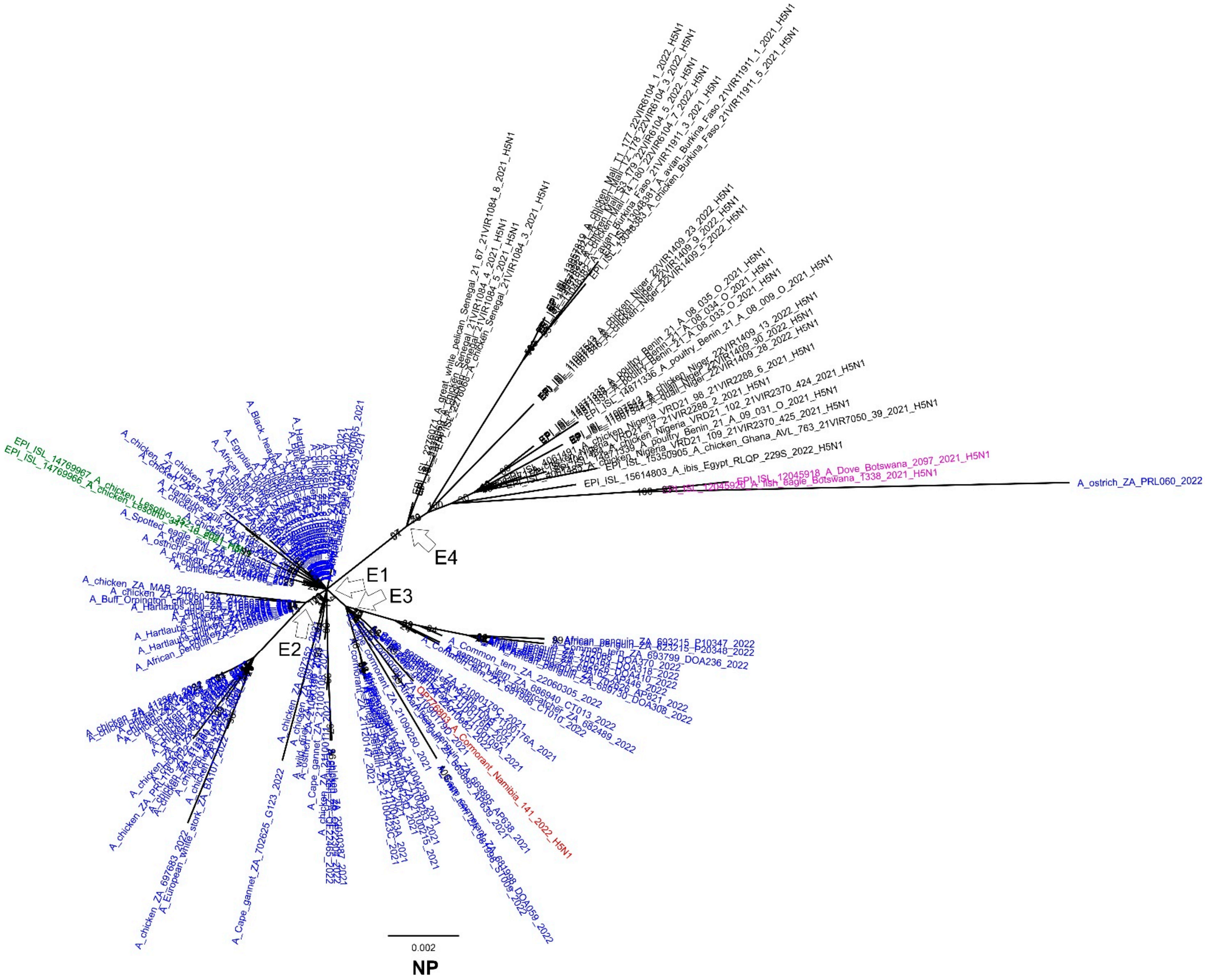
E1
E2
E3
E4
A_ostrich_ZA_PRL060_2022
EPI_ISL_15614803_A_ibis_Egypt_RLQP_229S_2022_H5N1
EPI_ISL_12045918_A_Dove_Botswana_2097_2021_H5N1
A_Cape_gannet_ZA_702625_G123_2022
0.002
NP

3(f)

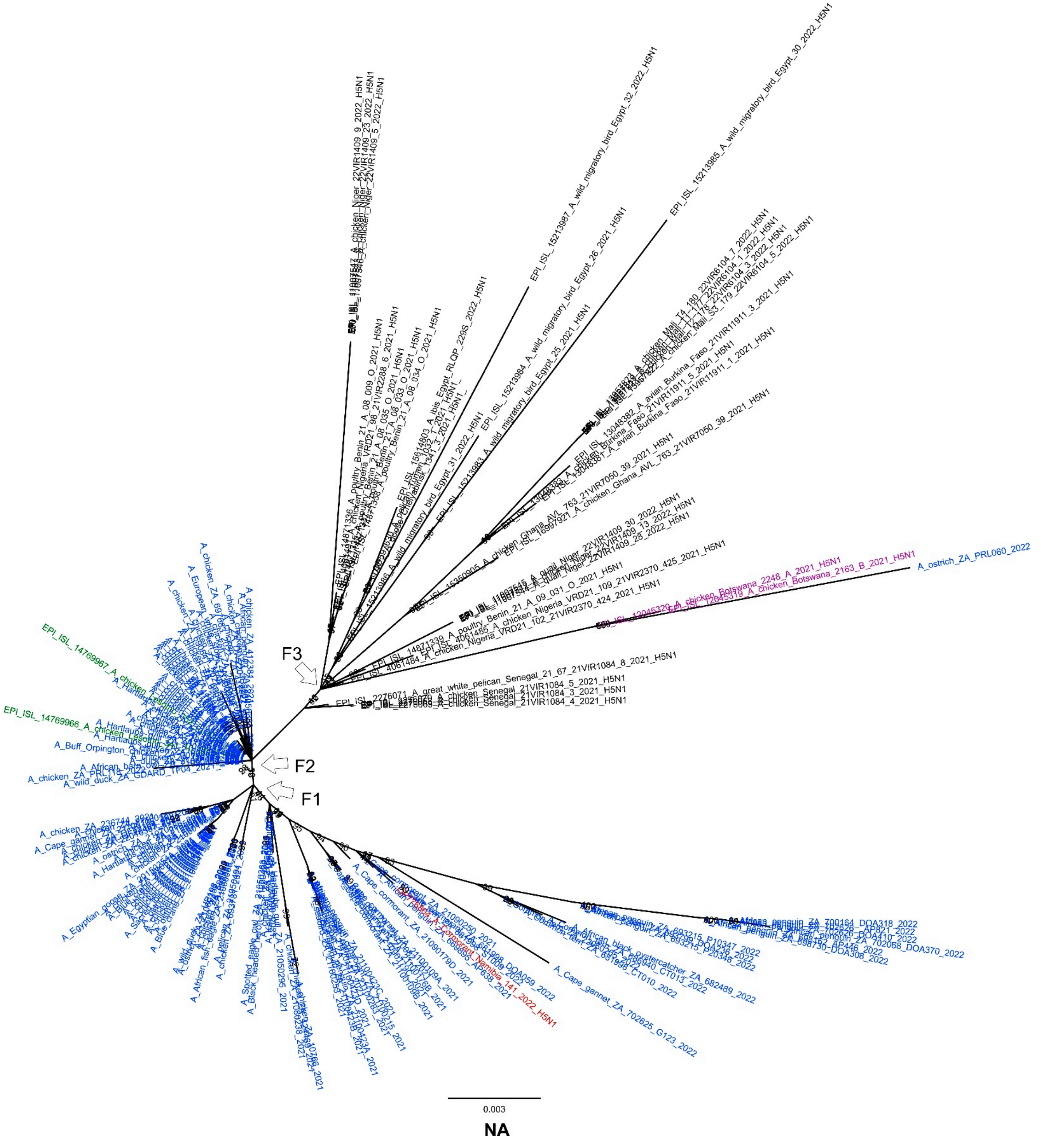
F1
F2
F3
0.003
NA
EPI_ISL_15213985_A_wild_migratory_bird_Egypt_30_2022_H5N1
EPI_ISL_15213987_A_wild_migratory_bird_Egypt_32_2022_H5N1
A_ostrich_ZA_PRL060_2022
A_great_white_pelican_Senegal_21VIR1084_5_2021_H5N1
A_Cape_gannet_ZA_702625_G123_2022
A_Buff_Orpington
A_wild_duck_ZA_GDARD_TF04_2021
A_chicken_ZA_PRL114_2021
A_cormorant_Namibia_141_2022_H5N1

3(g)

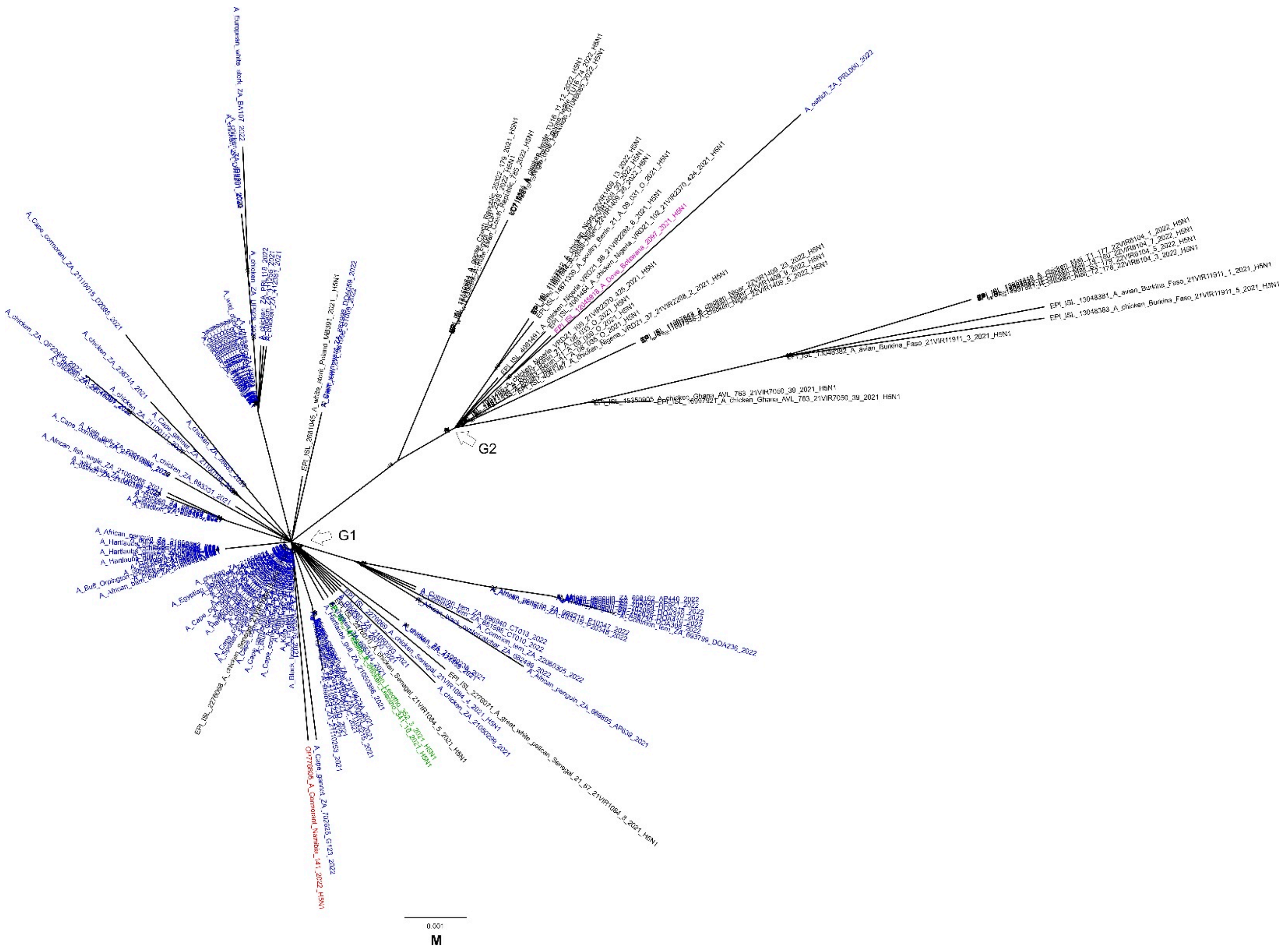

3(h)

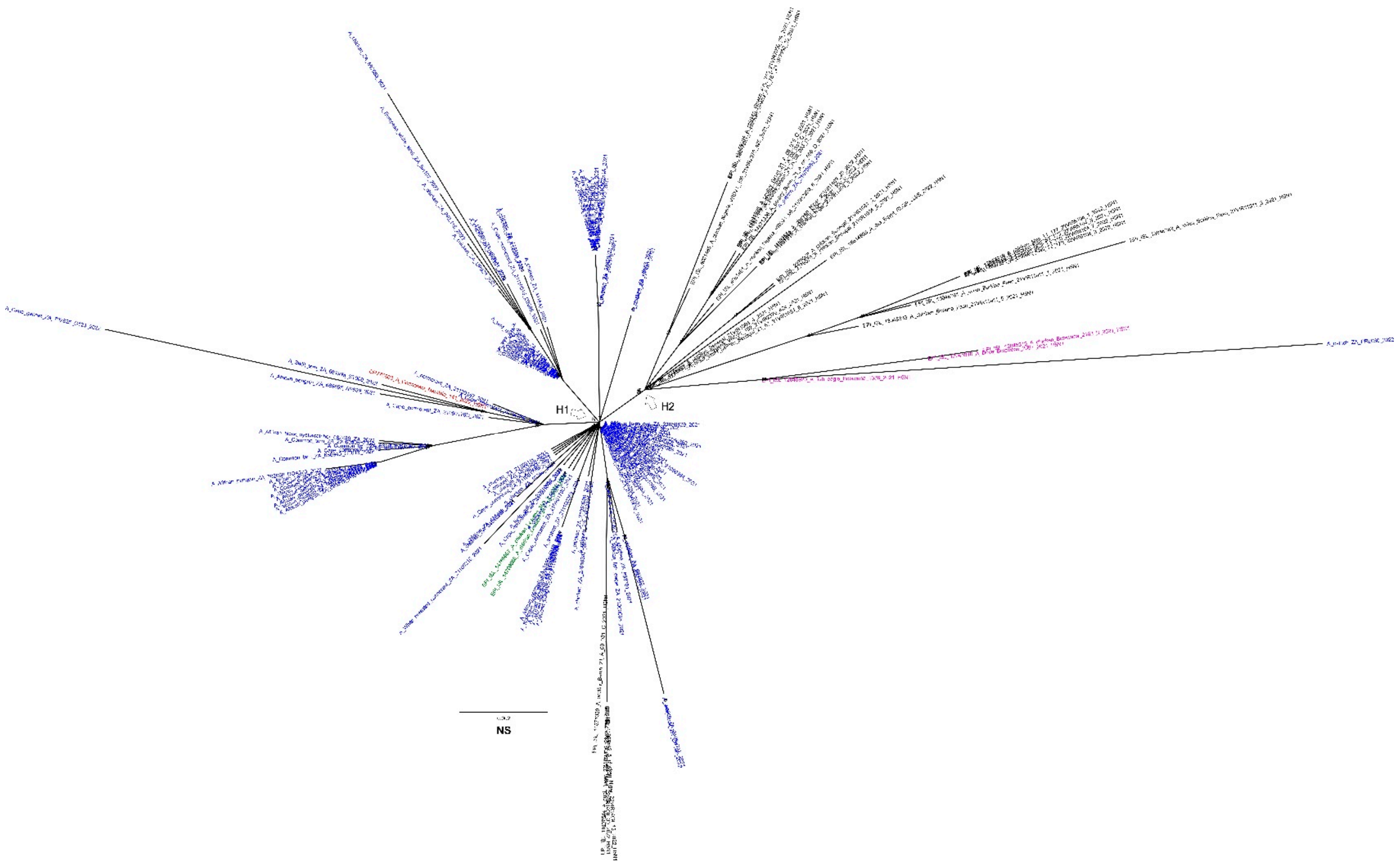

Figure S3a–h. Radial views of the Maximum Likelihood phylogenetic trees of the individual genome segments of H5N1 HPAI viruses used to identify subclades, as shown by the arrows. Letters A to H were used for the PB2, PB2, PA, HA, NP, NA, M and NS genes, respectively. South African viruses are highlighted in blue, Namibia in red, Lesotho in green and Botswana in magenta.
