# Supplementary figures and images for: The Molecular Epidemiology of Clade 2.3.4.4B H5N1 High Pathogenicity Avian Influenza in Southern Africa, 2021–2022
Source: Viruses. 2023 Jun 16;15(6):1383. doi: 10.3390/v15061383 (PMC10302261; doi:10.3390/v15061383)

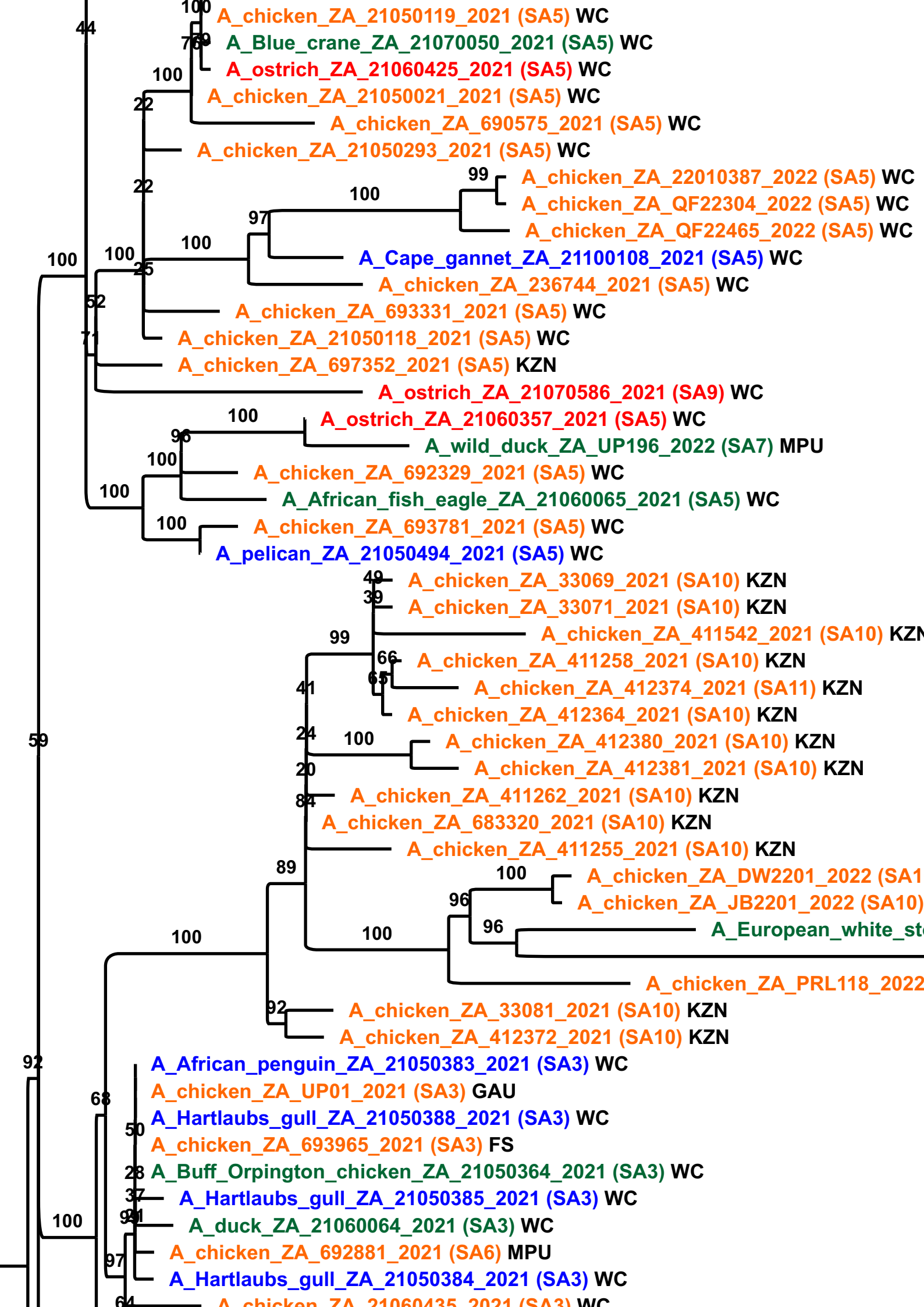

Supplement: Supplementary file 1 [file viruses-15-01383-s001.zip › Enlarged view of Figure 3.pdf]

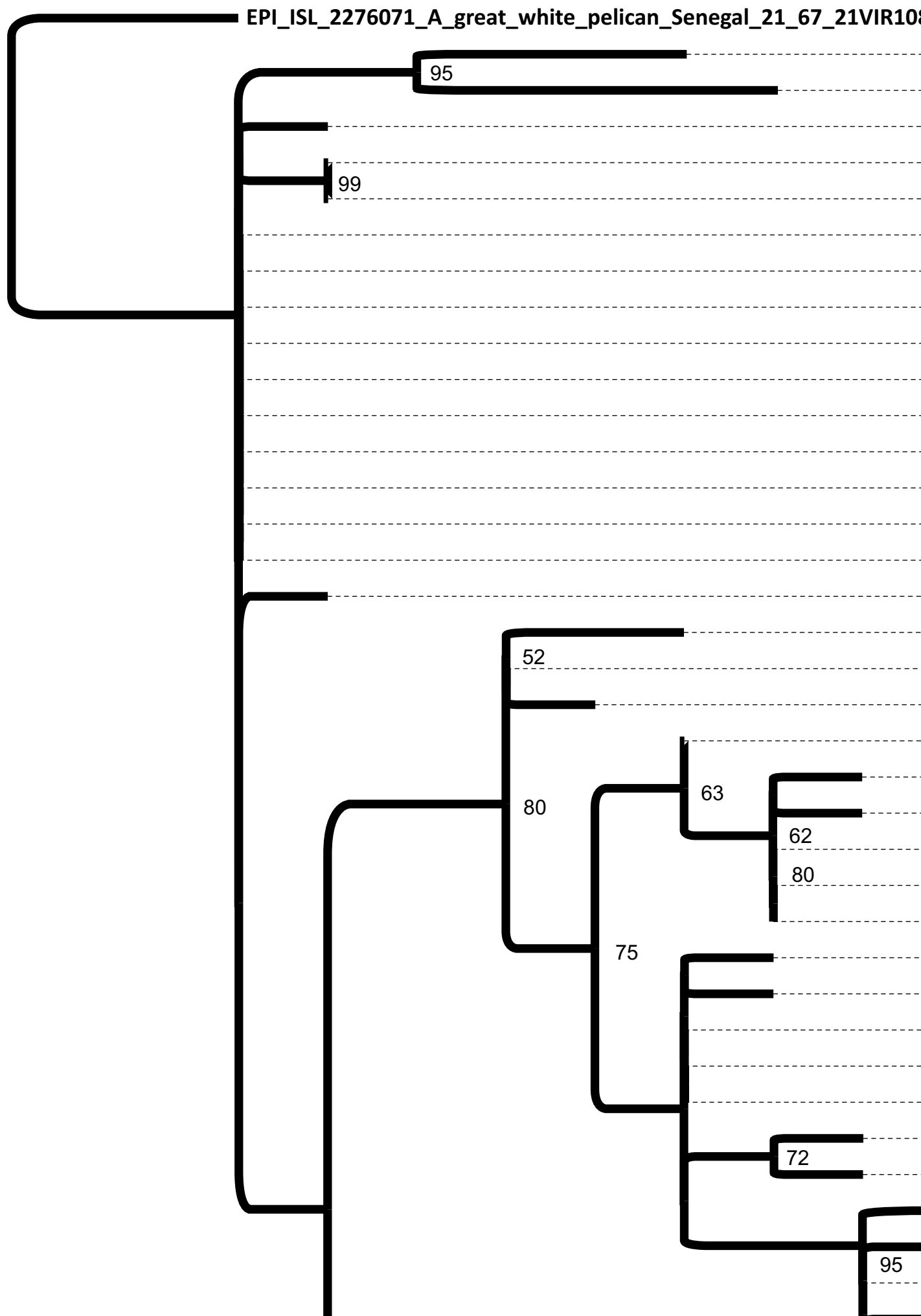

Supplement: Supplementary file 1 [file viruses-15-01383-s001.zip › Enlarged view of Figure 5.pdf]

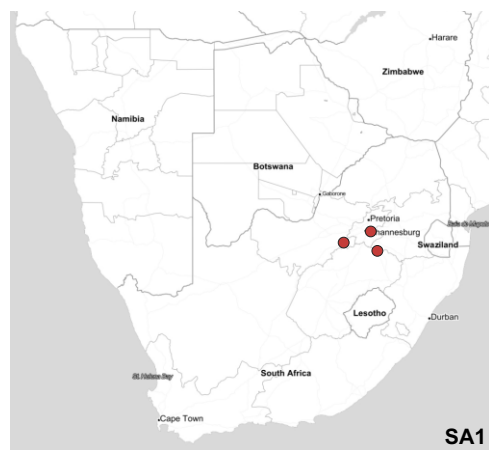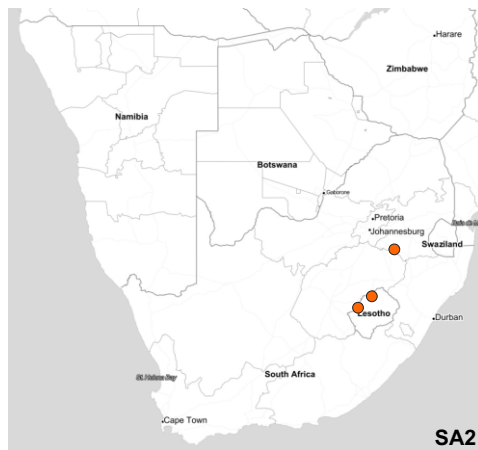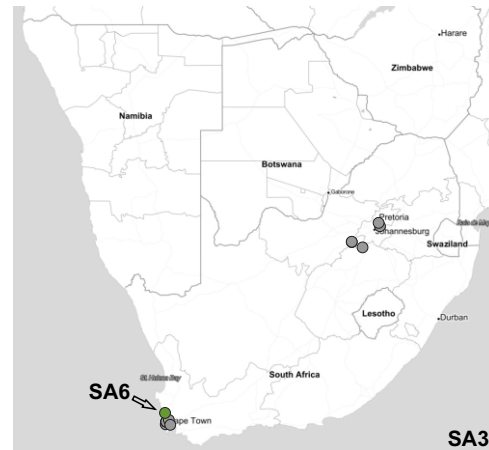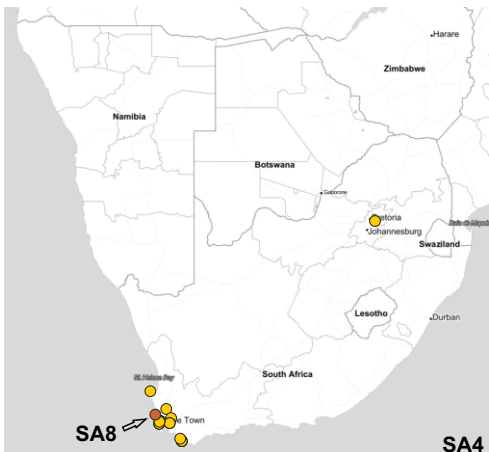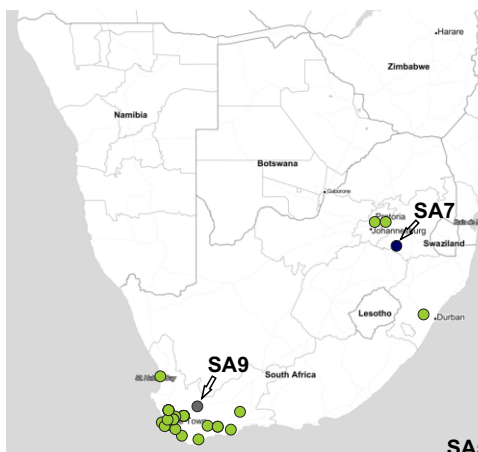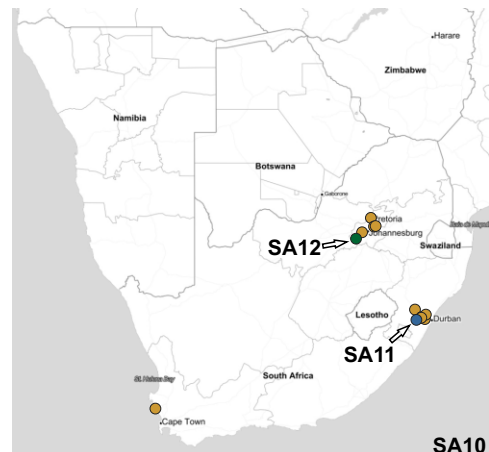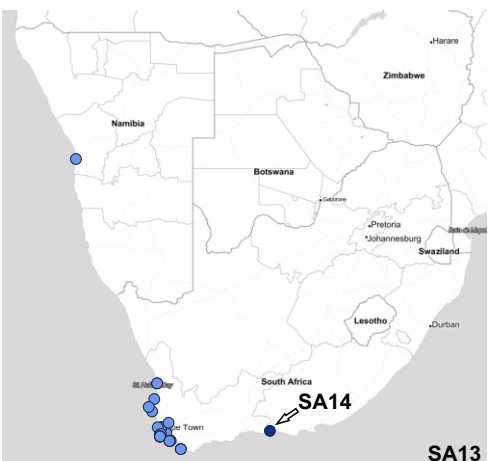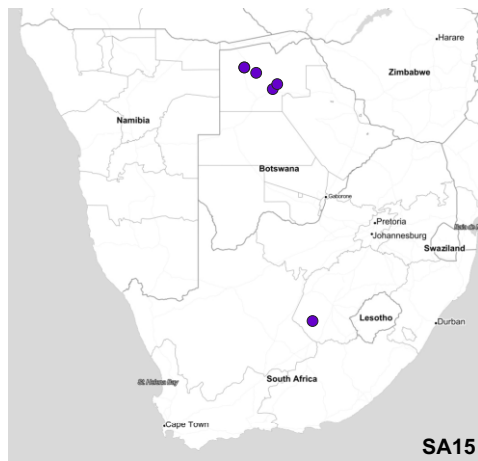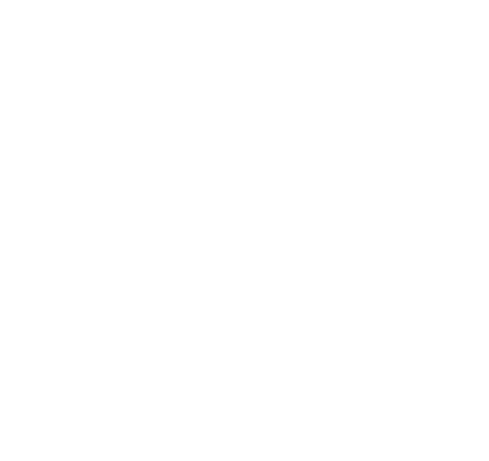

Supplement: Supplementary file 1 [file viruses-15-01383-s001.zip › Enlarged view of Figure 7.pdf]

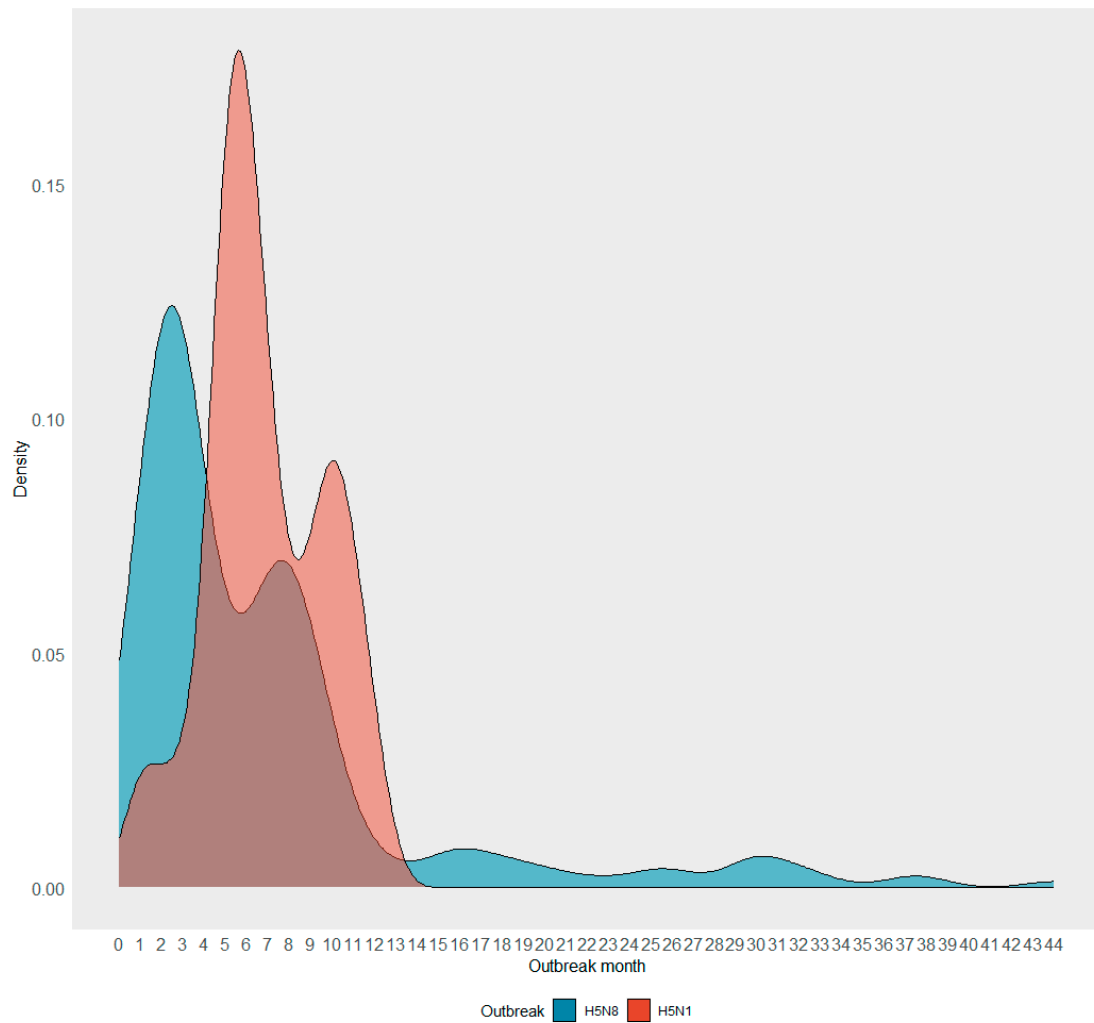

**Figure S2.** Density plot of HPAI outbreaks in South Africa by month.

Supplement: Supplementary file 1 [file viruses-15-01383-s001.zip › Figure S2.pdf]
